# System of Diseases of the Eye

**Published:** 1897-03

**Authors:** 


SYSTEM OF DISEASES OF THE EYE. By American, British, Dutch, French, German and Spanish authors. Edited by WILLIAM F. MORRIS, A. M., M. D., and CHARLES A. OLIVER, A. M., M. D., of Philadelphia, Pa. Volume I. Embryology, Anatomy and Physiology of the Eye. Octavo, pp. xvi.—670. With twenty-three full-page plates and 362 text illustrations. Philadelphia : J. B. Lippincott Co. 1896.

A long time has elapsed since the announcement of the preparation of this work. At last the first volume has made its appearance. The original plan was to issue the " system " in two volumes, but that has been changed, and it is now being published in four. The purpose of the editors is to cover in a thorough, but not too diffuse manner, the whole field of ophthalmology, both theoretically and practically. They have associated with them many able collaborators, and hope to have produced a work " which will take the place in the English language similar to that occupied by the Handbuch of Graefe and Saemisch in German and by the Traité Complet of de Wecker and Landolt in French." How well they have succeeded can be judged better when the work is completed. This first volume, however, is a splendid contribution to ophthalmology. It is not encumbered by a mass of useless material, but is solid meat from first to last.

The first section is on the Development of the eye, and is written by John A. Ryder, Ph. D., professor of comparative embryology in the University of Pennsylvania. It is a good résumé of the subject and occupies seventy pages of the text.

The anatomy of the orbit and the appendages of the eye constitutes the next section of thirty-eight pages. It is a most clear and instructive description by Dr. Thomas Dwight, Parkman professor of anatomy in Harvard University. Much information is presented here which one would look a long way to find elsewhere.

The anatomy of the eyeball and of the intraorbital portion of the optic nerve is quite an extended section of 108 pages, and is written by Dr. Frank Baker, professor of anatomy in the University of Georgetown. Like Prof. Dwight's article, it enters into a detailed description of the parts of which it treats and embodies a vast amount of information, some of which is not otherwise easily accessible. Dr. Baker has deviated from the plan of the other writers and introduced in foot-notes the synonyms, and oftentimes the etymology, of the anatomical words and terms used.

The largest section of the book is the next one, embracing 166 pages. In it Dr. George A. Piersol, professor of anatomy in the University of Pennsylvania, describes at length The microscopical anatomy of the eyeball. The subject is handled by a master-hand and the result is well-nigh perfect.

The anatomy of the intracranial portion of the visual apparatus comes next, and is treated by Dr. Alex. Hill, master of Downing College, etc., Cambridge, England. Within the space of thirty-four pages he gives the most recent facts and views in regard to the cerebral visual apparatus.

The next forty-two pages are devoted to Congenital malformations and abnormalities of the human eye. This section is contributed by William Lang, F. R. C. S., and E. Treacher Collins, F. R. C. S., both well-known ophthalmologists of London. It is an excellent résumé of congenital defects of the eye.

Dr. Edward Jackson, professor of diseases of the eye in the Philadelphia Polyclinic, contributes the following section on The dioptrics of the eye. In forty-six pages he gives the principles of reflection and refraction of light as applied to the eye. The different states of refraction of the eye, both with and without accommodation, are described and the means of correcting deviations from the emmetropic condition are briefly touched upon. Fuller discussion of the treatment of errors of refraction is reserved for a future volume. Dr. Jackson here, as always, writes clearly, comprehensively and to the point.

The next section takes up the subject of The perception of light. The author, J. McKeen Cattell, M. A., Ph. D., professor of experimental psychology in Columbia College, occupies thirty-four pages, and enters into consideration of some of the more abstruse phases of physiology, which pertains to the perception of light. He deals with the intensity of light, the perception of small differences, the comparison of magnitudes, methods for studying the accuracy of perception, intensity in relation to color, sharpness of sight, field of vision, fatigue, positive after-images, negative after-images, etc.

The section following, constituting forty-two pages, is also more or less abstruse, and is contributed by Dr. Eugen Brodhun, of Berlin, Germany. It treats of binocular vision, confliction of the field of vision, apparent and natural size of objects. Perception of depth in monocular vision and in binocular vision, binocular or stereoscopic vision, the horopter, and the like.

The next section is Normal color perception and is written by Dr. William Thomson, professor of ophthalmology in the Jefferson Medical College, assisted by Dr. Carl Weiland, clinical assistant in the Jefferson Medical College Hospital. This is a well-written chapter and the authors may be considered as authorities on the subject with which they deal. They accept the Young-Helmholtz theory of color vision.

The concluding section of thirty-four pages is written by Dr.

Carl Mays, of the University of Heidelberg, and is a learned presentation of the Photo-chemistry of the retina.

As will be seen by the above presentation of the divisions and authorship of this volume, the elementary foundations of ophthalmology are broadly and solidly laid. Each section is almost a treatise in itself. The work is elaborately and beautifully illustrated and put up in the publishers' highest art. It merits in every way the unstinted praise of critics, and should be sought by every careful student of ophthalmology. A. A. H.

